# Reduced length of intensive care unit stay and early mechanical ventilator weaning with enhanced recovery after surgery (ERAS) in free fibula flap surgery

**DOI:** 10.1038/s41598-023-50881-z

**Published:** 2024-01-03

**Authors:** Wei-Ling Hsiao, Yao-Cheng Wu, Hao-Chih Tai

**Affiliations:** 1 School of Nursing, National Taiwan University College of Medicine, Taipei, Taiwan; 2 Department of Nursing, National Taiwan University Hospital, Taipei, Taiwan; 3 Department of Surgery, National Taiwan University Hospital, National Taiwan University College of Medicine, 7 Zhong-Shan South Road, Taipei, 10002 Taiwan

**Keywords:** Medical research, Signs and symptoms

## Abstract

This study aimed to evaluate the effects of the enhanced recovery after surgery (ERAS) program on postoperative recovery of patients who underwent free fibula flap surgery for mandibular reconstruction. This retrospective study included 188 patients who underwent free fibula flap surgery for complex mandibular and soft tissue defects between January 2011 and December 2022. We divided them into two groups: the ERAS group, consisting of 36 patients who were treated according to the ERAS program introduced from 2021 to 2022. Propensity score matching was used for the non-ERAS group, which comprised 36 cases selected from 152 patients between 2011 and 2020, based on age, sex, and smoking history. After propensity score matching, the ERAS and non-ERAS groups included 36 patients each. The primary outcome was the length of intensive care unit (ICU) stay; the secondary outcomes were flap complications, unplanned reoperation, 30-day readmission, postoperative ventilator use length, surgical site infections, incidence of delirium within ICU, lower-limb comorbidities, and morbidity parameters. There were no significant differences in the demographic characteristics of the patients. However, the ERAS group showed the lower length of intensive care unit stay (ERAS vs non-ERAS: 8.66 ± 3.90 days vs. 11.64 ± 5.42 days, *P* = 0.003) and post-operative ventilator use days (ERAS vs non-ERAS: 1.08 ± 0.28 days vs. 2.03 ± 1.05 days, *P* < 0.001). Other secondary outcomes were not significantly different between the two groups. Additionally, patients in the ERAS group had lower postoperative morbidity parameters, such as postoperative nausea, vomiting, urinary tract infections, and pulmonary complications (*P* = 0.042). The ERAS program could be beneficial and safe for patients undergoing free fibula flap surgery for mandibular reconstruction, thereby improving their recovery and not increasing flap complications and 30-day readmission.

## Introduction

In 1975, Taylor et al. proposed the first vascularized fibular bone graft for lower limb reconstruction^1^. In 1989, Hidalgo expanded the indications for fibular free tissue transfer for reconstructing mandible compound defects^2^. In 1994, Wei and their team detailed the application of the osteoseptocutaneous fibula flap in the reconstruction of composite mandibular and soft tissue defects^3^. Kuo and colleagues chimed soleus muscle via muscular perforator with fibula osteoseptocutaneous flap for dead space obliteration^4^. Consequently, the fibula osteocutaneous flap has become a highly versatile choice of head and neck reconstruction.

Fibular bone can be used in reconstruction in various ways, including cortical autografts, bone allografts, vascularized bone grafts, fibular osteocutaneous flaps, and fibular osteomuscular flaps^5,6^. Among these options, free vascularized fibular grafting has gained prominence as it provides immediate mechanical support and has the potential for growth or hypertrophy, according to the patient’s condition.^7^ However, the occurrence of complications is influenced by various perioperative factors: anesthesia-related complications (nausea and vomiting, respiratory depression, aspiration pneumonia, pulmonary embolism, and urinary tract infection); intraoperative complications (improper fixation, neurovascular bundle damage, graft length discrepancy, or bleeding); and postoperative complications (graft fracture, surgical site infection, gait disturbance due to pain, equine deformity of the ankle, amputation, nerve injury with sensory loss or motor deficit or anastomosed vessel thrombosis, and partial or complete flap failure)^8–14^. To improve the quality of life of patients and reduce complications, it is crucial to optimize perioperative management.

In 1997, Kehlet first introduced the concept of enhanced recovery after surgery (ERAS) to promote quick patient recovery, restore normal activity levels, and reduce the length of hospital stay after surgery, leading to a decrease in complication rates and hospitalization costs^15^. Subsequently, many specialties have adopted ERAS programs, which have become the standard of care for several surgical procedures^16–24^, as confirmed by various systematic reviews and meta-analyses^25–33^. However, to the best of our knowledge, no studies have evaluated the impact of ERAS focusing solely on patients undergoing free fibula flap surgery for mandibular reconstruction^34^. As prospectively performing a randomized controlled trial with a sufficient number of patients undergoing high-risk surgery is challenging, a propensity score matching (PSM) analysis can reduce confounding and increase the level of evidence. Therefore, this novel study was conducted to assess the feasibility, safety, and efficacy of an adapted ERAS program in patients undergoing free fibular flap surgery using a PSM analysis.

## Materials and methods

### Design, participants and analysis

We conducted a retrospective screening at our single center, applying inclusion criteria for individuals who underwent free fibula flap surgery for complex mandibular and soft tissue reconstruction performed by the plastic surgical team in our institute between January 2011 and December 2022. Patients those with incomplete demographic data were excluded from the study. This retrospective study included 188 patients during this period. Patients were eligible for inclusion in the study if they underwent mandibular reconstruction using a free fibular flap. The ERAS group comprised 36 cases enrolled between January 2021 and December 2022, while the non-ERAS group consisted of 152 cases enrolled between January 2011 and December 2020. The plastic surgery team provided care to patients in both groups.

We evaluated the patient data, comparing patient demographics, anesthetic and operative data as shown in Table 1. The results indicate that three variables exhibit significant statistical differences (*P* < 0.05): age, gender, and smoking status. To reduce confounding variables, we employed the PSM method to match these 188 patients based on age, sex, and smoking status, with a caliper width of 0.1 and standard deviation of the propensity score (Table 1). The matching used a 1:1 ratio greedy nearest-neighbor matching approach, with a maximum propensity score difference of ± 1%. We calculated the standardized mean difference for each matched variable to determine PSM success. Ultimately, in this study, we first used the Kolmogorov–Smirnov test to investigate whether continuous variables followed a normal distribution. The analysis results, as shown in Table 1, indicate that Age (KS statistic = 0.138, *p* < 0.01), Operative time (KS statistic = 0.108, *p* < 0.05), Intraoperative blood loss (KS statistic = 0.238, *p* < 0.001), ICU length of stay (KS statistic = 0.247, *p* < 0.001), and Postoperative ventilator (KS statistic = 0.328,* p* < 0.001) all reached significance. This implies that Age, Operative time, Intraoperative blood loss, ICU length of stay, and Postoperative ventilator do not follow a normal distribution. Therefore, for subsequent comparisons of mean differences between different groups (ERAS Group and Non-ERAS Group) in the above-mentioned variables, we utilized the Mann–Whitney *U* test. Categorical variable differences between the ERAS and non-ERAS groups were assessed using chi-square or Fisher’s exact tests.

**Table 1:** Patient demographics and anesthetic and operative data.

	Before matching			After matching		
	ERAS group	Non-ERAS group	*P* value	ERAS group	Non-ERAS group	*P* value
Sample size	*n* = 36	*n* = 152		*n* = 36	*n* = 36	
Age, years	56.00 ± 14.89	61.97 ± 12.87	0.01*	56.00 ± 14.89	55.69 ± 12.21	0.51
Sex, male/female	29/7	98/54	0.04*	29/7	25/11	0.28
Smoker	34	120	0.02*	34	32	0.67^a^
KPS						
80–100	29	127	0.65	29	30	0.76
50–70	7	23		7	6	
0–40	0	2		0	0	
Diagnosis						
Cancer	27	115	0.79	27	29	0.57
Non-cancer	9	37		9	7	
ASA grade						
I	0	0	0.37	0	0	0.36
II	8	28		8	5	
III	28	124		28	31	
IV	0	0		0	0	
Operative time, minutes	902.31 ± 130.98	888.52 ± 255.31	0.65	902.31 ± 130.99	836.39 ± 229.79	0.06
Intraoperative blood loss, mL	298.61 ± 169.23	299.45 ± 189.20	0.39	271.33 ± 128.49	298.61 ± 169.24	0.55

Values are reported as numbers or mean ± SD.

*SD* standard deviation, *ERAS* enhanced recovery after surgery, *BMI* body mass index (weight (in kilograms) ÷ height (in meters)), *ASA* American.

^a^Fisher’s exact test.

For statistical analyses, a *P*-value < 0.05 was considered significantly different. PSM and statistical analyses were performed using SPSS software (version 26.0; SPSS, Inc., Chicago, Illinois, USA). This study was approved by the National Taiwan University Hospital Research Ethics Committee (approval number: 202302021RIN). The requirement for written informed consent was waived owing to the retrospective nature of the study.

### Outcome measures

The primary outcome measure was the length of ICU stay; secondary outcomes included flap complications, such as total flap loss, unplanned reoperation within 30 days, 30-day readmission, postoperative ventilator use length, vasopressor use, surgical site infections, incidence of delirium within ICU and lower limb comorbidities^13,14,35^, including peroneal nerve injury-induced sensory loss or foot drop. Other morbidity parameters include postoperative nausea and vomiting (PONV), urinary tract infections, and pulmonary complications. All data were collected from electronic medical records.

### ERAS and non-ERAS intervention

When ERAS programs were introduced in our hospital, there were no clear guidelines for free fibular flap surgery. Therefore, we adopted and improved the general ERAS principles^31–33^ and established ERAS programs at our hospital. In the preoperative phase, highlighting significant differences in the approach towards patient preparation. The ERAS group engages in clinical shared decision-making, consults physiotherapists, and conducts nutritional assessments using the MUST score, with interventions applied to those at severe nutritional risk. Another remarkable contrast is noted in the dietary restrictions before surgery; the ERAS group is allowed carbohydrate drinks closer to the surgery time compared to the strict fasting program of the non-ERAS group. In the perioperative phase, both groups adhere to similar programs regarding organ function evaluations and the prevention of antibiotic use, intraoperative safety checks, and anesthesia. Both groups difference in the specificities in liquid management and temperature management.

The postoperative comparison variances in patient care. The ERAS group employs multimodal analgesia, ensuring diversified pain management compared to the non-ERAS group's reliance on opioid analgesia. The approach to airway management is also more structured in the ERAS group, focusing on early withdrawal of ventilator use within 24 h. Prophylactic measures against postoperative nausea and vomiting in the ERAS group, flap monitoring, and advanced donor site and drain management techniques, such as negative pressure wound therapy, exemplify a more intricate and preemptive care strategy. Delirium prevention in the ERAS group involves the routine use of dexmedetomidine and early mobility. Furthermore, nutritional support and early mobilization programs in the ERAS group indicate a more active and planned recovery phase, involving sequential enteral nutrition treatment after awakening and bedside mobility from POD 1. Lastly, both groups receive education before discharge. Detailed variations in the postoperative programs for both groups are presented in Table 2.

**Table 2:** Comparison of programs between the ERAS and non-ERAS groups.

Program list	ERAS group	Non-ERAS group
Preoperative		
Clinical shared decision-making of ERAS	Yes	No
Consult Physiotherapist	Apply posterior ankle–foot-orthosis Education isometric and isotonic exercise on the bed	After transfer to the general ward
Nutritional assessment and intervention	Use MUST Score, patients with severe nutritional risk should be provided enteral nutrition supportive treatment	No
Organ function evaluation	Yes, check hematological, renal, kidney, liver, and coagulation function	Yes, similar to the ERAS group
Fasting and abstinence from drinking	Eating was allowed up to 6 h before surgery, and carbohydrate drinks were allowed to be consumed up to 2 h before surgery	Fasting and drinking for 8 h before the operation
Intraoperative		
Prevention of antibiotic use	Yes, include both aerobic and anaerobic bacteria	Yes, include both aerobic and anaerobic bacteria
Liquid management	Keep input/output balance Maintain urine output 0.5–1 cc/kg/h	No
Intraoperative safety check	Use time-out checklist	Use time-out checklist
Anesthesia	General anesthesia	General anesthesia
Temperature management	Maintain the core temperature of the patient not less than 36 °C (preheating fluid replenishment, thermal blanket, heater)	No
Postoperative		
Postoperative analgesia	Multimodal analgesia (Opioid, NSAIDs, acetaminophen, COX-2 inhibitors, α-2 agonists) Patient-controlled intravenous analgesia maintained until POD 2 or POD 3	Opioid analgesia
Airway management (SpO_2_ should be maintained above 90%)	Tracheostomy with ventilator support and structure weaning program by RT, early withdrawal ventilator use within 24 h	Tracheostomy with ventilator, weaning by RT, or Physician decision
Postoperative nausea and vomiting prevention	5-HT3 receptor antagonists are used prophylactically for the first 24 h	No
Flap monitoring	Monitor of color, temperature, and Doppler of recipient site by physician order	Similar to ERAS group
Donor site and drain management	Negative pressure wound therapy, early tube removal (latest POD 5)	Gauze cover Routine indwelling before discharge
Delirium prevention	Routing use dexmedetomidine and avoid midazolam	Use midazolam or Physician decision
Postoperative nutritional support	Sequential EN treatment after awakening	Gradually start EN after awakening
Early mobilization	Bedside ambulation from POD 1 (head up → limb movement isometric and isotonic exercise on the bed) Use posterior ankle–foot-orthosis	Ambulation on transfer to the general ward
Education before discharge	Yes	Yes

*MUST* malnutrition universal screening tool, *NSAIDs* non-steroidal anti-inflammatory drugs, *COX-2* cyclooxygenase-2, *POD* postoperative day, *RT* respiratory therapist, *5-HT3* selective serotonin receptor, *EN* enteral nutrition, *SpO*_*2*_ oxygen saturation.

### Ethics approval

The work described has been carried out in accordance with the Declaration of Helsinki (Code of Ethics of the World Medical Association for experiments involving humans). The data used in our study was approved by the Research Ethics Committee of the National Taiwan University Hospital (number 202302021RIN).

## Results

### Patients characteristics

After PSM, the present study included a total of 72 patients. There were 36 patients in the ERAS group (29 men and 7 women; mean age, 56 ± 14.89 years) and 36 patients in the non-ERAS group (25 men and 11 women; mean age, 55.69 ± 12.21 years). The baseline demographic characteristics and operative details of the two groups are compared in Table 1. A comparison of the demographic data revealed no significant intergroup differences. No significant differences in operative time or intraoperative blood loss were observed between the ERAS and non-ERAS groups. The Karnofsky Performance Status Scale (KPS) was used for the assessment of preoperative physical function and revealed no significant differences between the two groups (*P* = 0.76). A KPS score of 0–40 was observed in 0 patients, a KPS score of 50–70 was found in 13 patients (7 patients in the ERAS group vs. 6 patients in the non-ERAS group), and a KPS score ≥ 80 was found in 59 patients (29 patients in the ERAS group vs. 30 patients in the non-ERAS group). No significant differences in preoperative physical status were observed between the two groups (Table 1).

### Compliance with the ERAS program

Our ERAS program included 19 component interventions, and the overall program compliance was 91.2% (Table 3). The twelve interventions were used in 100% of the cases in the ERAS group, with a 100% compliance rate. The items with relatively low compliance were donor site and drain management (61.1%), early mobilization (61.1%), consult physiotherapist (75%), PONV prevention (77.8%), delirium prevention (83.3%), and clinical shared decision-making and education of ERAS (86.1%).

**Table 3:** Compliance with ERAS program or items.

ERAS items	Number (%)
Preoperative ERAS items	92.2%
Clinical shared decision-making and education of ERAS	31 (86.1)
Consult physiotherapist	27 (75)
Nutritional assessment and intervention	36 (100)
Organ function evaluation	36 (100)
No prolonged fasting	36 (100)
Intraoperative ERAS items	100%
Antimicrobial prophylaxis	36 (100)
Fluid management	36 (100)
Intraoperative safety check	36 (100)
Multimodal Anesthesia	36 (100)
Maintenance of normothermia	36 (100)
Postoperative ERAS items	85.7%
Multimodal analgesia	36 (100)
Airway management (SpO_2_ should be maintained above 90%)	32 (88.8)
Postoperative nausea and vomiting prevention	28 (77.8)
Flap monitoring	36 (100)
Donor site and drain management	22 (61.1)
Delirium prevention	30 (83.3)
Early enteral feeding	36 (100)
Early mobilization	22 (61.1)
Education of self-care before discharge	36 (100)
Overall compliance (rate)	**91.2%**

*ERAS* enhanced recovery after surgery, *SpO*_*2*_ oxygen saturation.

### Primary and secondary outcomes

The primary outcome was the ICU length of stay. On average, the ERAS group stayed 8.86 days, while the non-ERAS group stayed 11.64 days, a statistically significant difference (*P* = 0.003). Unplanned reoperations were across both groups: 3 cases for ERAS and 4 for non-ERAS. The 30-day readmissions were also close, with 2 in the ERAS group and 1 in the non-ERAS group. Each group 3 instances of flap complications resulting in flap loss. A notable difference was observed in the duration of postoperative ventilator use; the ERAS group averaged 1.08 days, which is significantly shorter than the 2.03 days in the non-ERAS group (*P* < 0.001). With regards to vasopressors, 3 patients in the ERAS group were off them by the end of the surgery, in comparison to 7 patients in the non-ERAS group. The ERAS group had no instances of surgical site infections, while the non-ERAS group reported 3 cases.

The incidence of delirium was 3 patients (8.33%) from the ERAS group and 5 patients (or 13.89%) from the non-ERAS group (*P* = 0.710). The non-ERAS group had a higher incidence of lower limb comorbidities with 6 patients, compared to 2 from the ERAS group, though this difference wasn't statistically significant (*P* = 0.260). Postoperative morbidity parameters, such as PONV, urinary tract infections, and pulmonary complications, exhibited a significant decrease in the ERAS group. There was a statistically significant difference between the two groups for PONV (*P* = 0.041). However, the differences in urinary tract infection and pulmonary complications did not statistical significance, yielding *p*-values of 0.493 and 0.674, respectively. The overall morbidity parameters also demonstrated a statistically significant difference (*P* = 0.042). These outcomes are detailed in Table 4.

**Table 4:** Postoperative recovery and complications outcomes.

Outcome measure	ERAS group *n* = 36	Non-ERAS group *n* = 36	*P* value
ICU length of stay, days, mean ± SD	8.86 ± 3.90	11.64 ± 5.42	0.003**
Unplanned reoperation	3	4	1.000^a^
30-day readmission	2	1	1.000^a^
Flap complication (flap loss)	3	3	1.000^a^
Postoperative ventilator, days, mean ± SD	1.08 ± 0.28	2.03 ± 1.06	< .001***
Off vasopressors at end of surgery	33	29	0.173
Surgical site infections	0	3	0.239^a^
Incidence of delirium (%)	3 (8.33)	5 (13.89)	0.710^a^
Lower limb comorbidities	2	6	0.260^a^
Morbidity parameters			0.042^a^*
PONV	2	8	0.041*
Urinary tract infection (UTI)	0	2	0.493^a^
Pulmonary complications (VAP)	2	4	0.674^a^

*ERAS* enhanced recovery after surgery, *PONV* postoperative nausea, and vomiting, *SD* standard deviation.

^a^Fisher’s exact test.

## Discussion

The results showed that our ERAS program was beneficial and safe for patients undergoing free fibula flaps surgery. The ICU length of stay, postoperative ventilator use length, and morbidity parameters were lower in the ERAS group than those in the non-ERAS group, without any observed increase in 30-day readmission or flap complications. ERAS programs, has been instrumental in shortening the duration of ICU stays for patients. This achievement can be attributed to several key components of the ERAS programs. ERAS adopts a multimodal approach to care, addressing multimodal analgesia pain control, airway management, fluid regulation, nutritional support, and early mobilization. In the ERAS program, structured ventilator weaning refers to a carefully planned and gradual process of transitioning a patient from mechanical ventilation to spontaneous breathing. This weaning process protocol designed by respiratory therapist (RT) involves regular assessments, monitoring of respiratory parameters, and a step-by-step reduction in ventilator support as the patient's respiratory function improves. It follows a defined protocol to minimize complications, ensure a smooth transition and shorten the duration of mechanical ventilation. In the present study, a retrospective study of 16 ERAS programs for flap-based reconstruction found that ERAS principles have been widely and effectively applied to various flap types. A meta-analysis of 12 comparative studies (eight for breast reconstruction and four for head and neck reconstruction) found that ERAS programs significantly reduced LOS without affecting complication rates^34^. In this study, the patient demographic characteristics of the two groups were similar and the same surgical procedure was used, leading to no difference in the surgical or pathological results. However, the ERAS group had faster postoperative recovery and shorter ICU length of stays than the non-ERAS group, and these results were closely related to the ERAS program.

This study was based on three phases, and 19 core ERAS components. In our study, the compliance with ERAS was 91.2%. Furthermore, the intra-operative ERAS items had the highest compliance rates (100% for all items). Similar to previous study, the variance in compliance of individual ERAS components suggests the probability of high compliance rates (with an average above 90%)^38^. The items with relatively low compliance included donor site and drain management (61.1%), owing to the utilization of negative pressure wound therapy at the donor site. As this is not covered by health insurance and requires self-payment, patients with poor economic status are unable to afford its cost. Additionally, early mobilization revealed low compliance (61.1%). The low rate of early mobilization compliance following free fibula flap surgery in the past has been attributed to an increased emphasis on bed rest. This may have resulted in a lack of education of the medical team regarding the necessity and importance of lower-limb activity. ERAS represents a multifaceted perioperative management approach tailored to facilitate rapid postoperative rehabilitation. This program encompasses preoperative education, rehabilitation, nutritional assessment, and intervention prior to the operation, specific anesthesia techniques, multimodal analgesia, and an emphasis on early nutrition and mobility. However, challenges persist in the complete implementation of all its elements. Prior studies on ERAS in colorectal surgery have highlighted early mobilization and multimodal pain management as the most influential factors in reducing length of stay^39^. Moreover, readmissions tend to rise with an increase in morphine milligram equivalents (MME) per day. It would be beneficial for future studies to enlarge the sample size to analyze the efficacy of each ERAS items for fibula flap reconstruction surgery.

Based on the previous literature review, the primary distinction between the free fibula flap and other soft tissue-free flaps lies in the higher likelihood of complications such as drop foot and wound-related issues like infection and non-healing wounds^40,41^. Paresthesia and persistent lower-limb pain or discomfort are present in numerous patients. The present study reported long-term morbidities in 17% of patients, including leg weakness, ankle instability, great toe contracture, and decreased ankle mobility^41^. Currently, clinicians believe that the mechanism behind drop foot can be attributed to two factors: one is traction injury during fibula flap harvest, representing stage of neurapraxia, which generally recovers over time. The other is nerve injury, which may range from axonotmesis to neurotmesis, and recovery varies accordingly. Careful assessment of the patient's risk before surgery is important. Additionally, to reduce complications, measures for nerve protection during the procedure should be adopted. Ankle stiffness, ankle instability, transient peroneal motor loss, or sensory loss may occur after surgery^40^. Although donor site morbidity may not influence the outcome of major or life-threatening conditions, ankle stiffness may increase rehabilitation recovery time and influence the postoperative quality of life. In the ERAS group, immediate postoperative posterior ankle–foot-orthosis was applied to maintain the ankle at a 90-degree angle, preventing foot drop and ankle stiffness. In our study, the incidence of lower limb comorbidities was lower in the ERAS group (N = 3/36, 8.3%) compared with the non-ERAS group (N = 7/36, 19.4%), despite not having statistical significance. This suggests the need for longer-term follow-up, as immediate postoperative plaster fixation may assist in preserving the ankle at a 90-degree angle. This could potentially make rehabilitation easier compared to cases where the ankle drops to more than a 90-degree angle. While there is currently no specific literature addressing the prevention of drop foot complications during fibula flap harvest, we believe that this approach can contribute to improving the likelihood of late complications associated with drop foot in patients. Further studies with larger sample sizes are required to validate this finding.

However, this study had several limitations. This study had a retrospective design, the sample size was small, and the observation time was limited to the hospitalization period. Owing to the lack of long-term follow-up data, definitive conclusions cannot be drawn from these results. Furthermore, the ERAS and non-ERAS groups were assessed at different times, which may have introduced an analytical bias into the study. Hence, multicenter studies with larger cohorts, prospective studies, and long-term follow-ups are required to confirm the efficacy of our ERAS program in patients undergoing free fibula flap surgery for mandibular reconstruction.

## Conclusions

Although the ERAS program has achieved success globally, there are still many challenges to overcome. ERAS is a postoperative care option that requires continuous improvement and adaptation to different surgical methods and technologies. This approach emphasizes the integration of preoperative, intraoperative, and postoperative care. This study aimed to compare the short-term outcomes of patients who underwent free fibula flap surgery for mandibular reconstruction with and without the implementation of the ERAS using a PSM analysis. The findings suggest that the ERAS program can significantly reduce the intensive care unit LOS, postoperative ventilator use days, and morbidity parameters, without increasing postoperative complications and readmission rates. In our study, while the difference in the incidence of lower limb comorbidities did not attain statistical significance, we suggest that the immediate use of ankle–foot orthosis might aid in preventing drop foot and enhancing the success rate of rehabilitation. Nonetheless, additional long-term data are needed to validate this outcome. Therefore, the ERAS program can be a safe and beneficial option for patients undergoing free fibula flap surgery for mandibular reconstruction.

## Data Availability

The data underlying the findings presented in this report are available from the corresponding author upon reasonable request. For questions about data access or use, please contact taihc@ntu.edu.tw.
